# Inflammation centered muscle signature of sarcopenia from postmenopausal women in Shanghai China

**DOI:** 10.3389/fcell.2025.1726045

**Published:** 2025-12-18

**Authors:** Yongqian Fan, Shangjin Lin, Ming Ling, Tao Cui, Cong Chen, Fengjian Yang

**Affiliations:** 1 Department of Orthopaedic Surgery, Huadong Hospital Affiliated to Fudan University, Shanghai, China; 2 Department of Orthopaedic Surgery, The First Affiliated Hospital of Wenzhou Medical University, Wenzhou, Zhejiang, China

**Keywords:** sarcopenia, skeletal muscle, inflammation, RNA sequencing, random forest

## Abstract

**Introduction:**

Sarcopenia reflects age-related failure of muscle maintenance and regeneration, with chronic low-grade inflammation disrupting the satellite cell niche. Validated tissue biomarkers that connect inflammaging to clinical phenotypes remain limited. We sought to define an inflammation-centered muscle transcriptomic signature in postmenopausal women from Shanghai China and to assess its association with case–control status and muscle phenotypes.

**Methods:**

We prospectively enrolled 20 women undergoing femoral fracture surgery including sarcopenia (n = 8) and controls (n = 12). Vastus lateralis biopsies underwent RNA sequencing. Differentially expressed genes were identified and intersected with inflammation-associated genes from GeneCards. Random-forest models prioritized a minimal marker set. Discrimination was assessed with receiver operating characteristic analysis. Associations with appendicular lean mass, handgrip strength and calf circumference were tested.

**Results:**

We detected 301 differentially expressed genes enriched for p53, MAPK, TNF and NF-κB signaling together with ubiquitin-mediated proteolysis and cellular senescence. Intersection with the inflammation catalogue yielded 22 candidates that separated cases from controls by unsupervised clustering. Random-forest ranking nominated a five-gene panel JUN, SOCS3, CP, PTEN and C4B that showed strong single-gene discrimination with areas under the curve from 0.830 to 0.906. Higher expression of these genes was inversely associated with muscle quantity and strength.

**Discussion:**

This work is a pilot study that delineates an inflammation-associated transcriptomic phenotype of sarcopenia in postmenopausal women. These markers nominate testable inflammatory pathways and provide a rationale for validation studies designed to determine whether non-invasive surrogates or larger cohorts can substantiate their translational relevance.

## 1 Introduction

With the rapid growth of the aging population, the health burden attributable to age-related conditions has become a major public concern. Sarcopenia, a chronic disorder tightly linked to aging, is characterized by an imbalance between skeletal muscle protein synthesis and degradation, leading to a progressive decline in muscle mass and function (Yuan and Larsson, 2023). Although sarcopenia was formally recognized as a distinct disease entity in 2016 (Falcon and Harris-Love, 2017), its pathogenesis remains incompletely understood.

Multiple factors are implicated in primary sarcopenia, including chronic low-grade inflammation, excessive glucocorticoid exposure, mitochondrial dysfunction, loss of motor neurons associated with accelerated aging, exaggerated apoptosis, and reduced satellite cell activity (Morley et al., 2014). Among these, chronic low-grade inflammation is considered a key driver that promotes proteolysis, suppresses protein synthesis, and impairs myogenic regeneration, thereby accelerating the decline in muscle mass and performance (Bano et al., 2017). Besides, chronic low-grade inflammation reshapes the aged muscle stem cell (MuSC) niche through senescence-associated secretory programs and dysregulated immune-stromal crosstalk, thereby blunting MuSC activation, self-renewal, and effective regeneration (Tidball, 2017). Consistently, geriatric MuSCs show p16^INK4a^-driven geroconversion and impaired asymmetric division under regenerative pressure, mechanistically linking inflammaging to defective myogenesis (Sousa-Victor et al., 2014).

The advent of high-throughput transcriptomic profiling has provided powerful tools to elucidate disease mechanisms, discover diagnostic biomarkers, and nominate therapeutic targets. However, transcriptome studies of human muscle tissue from patients with sarcopenia remain relatively scarce, largely due to the challenges of obtaining muscle biopsies and the limited diffusion of omics technologies in this field. Focusing on the vastus lateralis of elderly women with sarcopenia, transcriptome sequencing can reveal differentially expressed genes (DEGs), enriched biological pathways, and inflammation-related expression programs associated with disease onset and progression.

Machine-learning methods can further prioritize robust, clinically informative gene signatures. In this study, we applied random forest (RF)—an ensemble algorithm that aggregates multiple decision trees to improve predictive accuracy and generalizability—to identify inflammation-related signature genes most strongly associated with sarcopenia (Kursa, 2014). RF is well suited to high-dimensional omics data and provides interpretable measures of feature importance for classification tasks.

Accordingly, we collected vastus lateralis samples from elderly women with sarcopenia and matched healthy controls for RNA-sequencing analysis. By integrating differential expression with RF-based feature selection, and coupling transcriptomic findings with clinical phenotypes of muscle mass and function, we aimed to (i) delineate inflammation-related transcriptional alterations and signaling pathways linked to sarcopenia and (ii) derive a concise inflammatory gene signature to prioritize pathways and markers for future validation and mechanistic study.

## 2 Methods

### 2.1 Participants

Between January and December 2023, we consecutively enrolled elderly women scheduled for open reduction and internal fixation (ORIF) of femoral fractures at Huadong Hospital, Fudan University. Twenty participants were included (sarcopenia, n = 8; non-sarcopenia, n = 12). Sarcopenia was defined per AWGS 2019 as appendicular lean mass index (ALM/height^2^) < 5.4 kg/m^2^ plus handgrip strength <18 kg in the dominant hand. All participant data were de-identified at the time of collection using study-specific codes; direct identifiers were removed. The protocol was approved by the Institutional Review Board (No. 2023K201), and written informed consent was obtained. Vastus lateralis biopsies were collected intraoperatively, snap-frozen in liquid nitrogen, and stored at −80 °C.

Inclusion criteria: postmenopausal women ≥60 years; provided consent; planned ORIF for femoral fracture.

Exclusion criteria: severe cardiac/hepatic/renal disease or malignancy; long-term corticosteroids/immunosuppressants; other primary myopathies (e.g., dystrophy, myositis); cognitive/psychiatric disorders precluding consent.

### 2.2 Minimum sample size

An *a priori* power analysis (R v4.1.3) targeting detection of ≥2-fold changes (|log2FC| ≥ 1) with α = 0.001 and β = 0.10 (power = 90%) indicated ≥6 participants per group. The final sample (8 and 12 per group) met and exceeded this threshold.

### 2.3 Clinical data collection

At admission, demographics, anthropometrics, medical history, and medications were recorded. Fasting venous blood drawn on day 2 provided CBC, CRP, albumin, calcium, and phosphorus. Calf circumference was measured on the non-injured leg at a standardized level 10 cm distal to the patella, with participants relaxed; a non-elastic tape was placed horizontally without compressing soft tissue.

### 2.4 Assessment of muscle mass and function

Whole-body dual-energy X-ray absorptiometry (DEXA) quantified limb muscle mass (appendicular lean mass index, ALM). Handgrip strength of the dominant hand was measured using a Jamar dynamometer (three trials, 3-min rest intervals). For analyses, the highest (maximum) value across the three trials was used.

### 2.5 RNA sequencing

Total RNA was extracted from intraoperative vastus lateralis biopsies under low-temperature conditions. RNA purity (spectrophotometry) and integrity (electrophoresis and fragment/integrity profiling) were assessed, and only quality-qualified samples proceeded to library preparation. Poly(A)-selected mRNA libraries were generated by controlled fragmentation, first- and second-strand cDNA synthesis, end repair with A-tailing, and adapter ligation, followed by bead-based cleanup and size selection to obtain a narrow insert distribution (∼300–500 bp). Libraries were amplified with limited PCR cycles, quantified fluorometrically, and profiled for insert-size distribution. Pooled libraries were sequenced on an Illumina platform (paired-end), with standard in-run quality metrics monitored to ensure depth and quality adequate for gene-level quantification.

### 2.6 Bioinformatics

1. Preprocessing and count matrix construction: Raw reads were demultiplexed, adapters and low-quality bases removed, and quality summarized. Reads were aligned to a current human reference genome assembly and summarized to gene-level counts to build an expression matrix; low-abundance genes were filtered to mitigate technical noise.
2. Normalization and differential expression: In R, library-size normalization and adjustment for technical variation were performed. Differential expression was computed with DESeq2 using the prespecified group design, yielding log2 fold-changes with multiple testing correction; significance thresholds were |log2FC| ≥ 1 and FDR ≤0.05. Volcano plots were produced with ggplot2.
3. Functional annotation and pathway enrichment: Using the set of expressed genes as background, clusterProfiler was applied for GO and KEGG enrichment, with significance determined by adjusted p values and visualized via bubble and bar plots.
4. Inflammation gene set and intersections: Genes associated with “inflammation” were retrieved from GeneCards with relevance score (RS) > 5 and intersected with DEGs to define inflammation-related DEGs. Venn diagrams and heatmaps (pheatmap) summarized overlap and expression patterns.
5. Feature selection and in-model validation: Random-forest models (randomForest package) were tuned for the number of trees and mtry to minimize out-of-bag (OOB) error. Features were ranked by Gini importance, and the top five were designated as inflammatory signature genes; group-wise expression differences were visualized with boxplots and heatmaps.
6. Discrimination and correlation analyses: Receiver operating characteristic (ROC) curves and AUCs (pROC) quantified single-gene discriminative performance (AUC >0.70 denoting acceptable discrimination). Optimal cutoffs were determined by Youden’s index with 95% confidence intervals (CI). Associations between signature-gene expression and muscle metrics were tested with cor. test (Pearson or Spearman as appropriate) and visualized using scatterplots.

### 2.7 Statistical analysis

Normality was examined with the Shapiro–Wilk test. Group comparisons used independent-samples t-tests for normally distributed data (mean ± SD) and Mann–Whitney U tests for non-normal data (median [IQR]). Linear regression analyzed relationships between continuous variables. In view of the limited sample size in this pilot study, multivariable regression was not performed to reduce the risk of overfitting and unstable parameter estimates. Multivariable modeling will be reserved for larger validation cohorts in the future. Two-sided *p* < 0.05 was considered statistically significant. All analyses were performed in R v4.1.3.

## 3 Results

### 3.1 Clinical characteristics of elderly women with and without sarcopenia

Participant characteristics are summarized in Table 1. The mean age was slightly higher in the sarcopenia group than in the non-sarcopenia group (73.88 ± 10.95 vs. 70.25 ± 4.97 years), but the difference was not statistically significant (*p* = 0.326), suggesting minimal confounding by age on downstream transcriptomic comparisons.

**TABLE 1:** Clinical characteristics of postmenopausal women with and without sarcopenia.

Clinical parameter	Sarcopenia (n = 8)	Non-sarcopenia (n = 12)	*P* Value
Age (years)	73.88 ± 10.95	70.25 ± 4.97	0.326
BMI (kg/m^2^)	23.11 ± 2.01	22.72 ± 2.81	0.741
RBC (10^12^/L)	3.64 ± 0.56	4.02 ± 0.59	0.177
WBC (10^9^/L)	5.55 (5.12, 7.17)	5.60 (5.20, 6.25)	0.999
Lymphocyte count (10^9^/L)	1.34 ± 0.35	1.52 ± 0.54	0.428
Monocyte count (10^9^/L)	0.43 (0.42, 0.47)	0.36 (0.31, 0.43)	0.176
Neutrophil count (10^9^/L)	3.96 (3.41, 5.43)	3.48 (2.75, 4.30)	0.384
CRP (mg/L)	14.52 (10.20, 32.85)	7.35 (4.66, 26.89)	0.521
Albumin (g/L)	40.62 ± 4.90	40.13 ± 3.37	0.793
Serum calcium (mmol/L)	2.20 ± 0.15	2.25 ± 0.08	0.302
Serum phosphorus (mmol/L)	1.07 ± 0.11	1.12 ± 0.17	0.539
ALM (kg/m^2^)	4.99 (4.80, 5.03)	5.79 (5.55, 7.00)	0.001
Handgrip strength (kg)	15.81 ± 2.05	18.73 ± 3.02	0.028
Calf circumference (cm)	30.35 ± 2.04	34.59 ± 2.70	0.002

Variables with normal distribution are presented as mean ± SD and compared using the independent-samples t-test; non-normal variables are presented as median (IQR) and compared using the Mann–Whitney U test. ALM, appendicular lean mass; CRP, C-reactive protein.

Body mass index (23.11 ± 2.01 vs. 22.72 ± 2.81 kg/m^2^) and serum albumin (40.62 ± 4.90 vs. 40.13 ± 3.37 g/L) were comparable between groups. Median CRP was higher in the sarcopenia group (14.52 mg/L) than in the non-sarcopenia group (7.35 mg/L), although this difference did not reach statistical significance (likely reflecting the limited sample size). Routine hematologic indices and serum calcium/phosphorus levels were similar across groups.

Measures of muscle status differed as expected. The median ALM was significantly lower in the sarcopenia group compared with the non-sarcopenia group (4.99 vs. 5.79 kg/m^2^; *p* = 0.001). Handgrip strength was also lower in participants with sarcopenia (15.81 ± 2.05 vs. 18.73 ± 3.02 kg), and calf circumference was markedly reduced (30.35 ± 2.04 vs. 34.59 ± 2.70 cm), with statistically significant between-group differences (Table 1).

### 3.2 DEGs in sarcopenia

**FIGURE 1:**
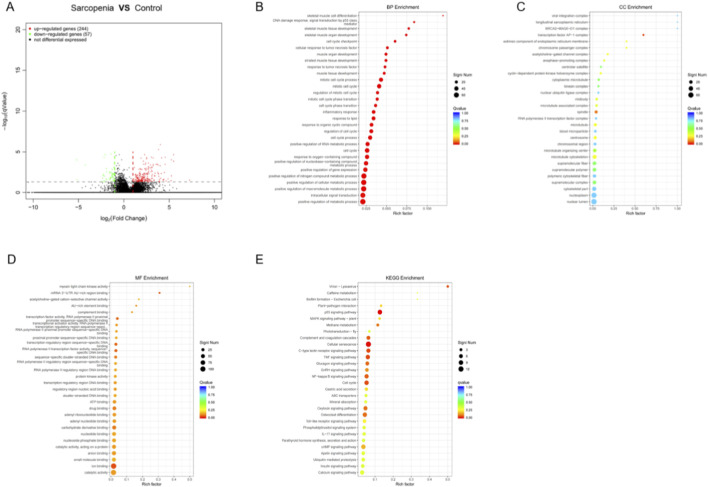
Transcriptomic alterations and pathway enrichment in sarcopenia. **(A)** Differentially expressed genes between sarcopenia and non-sarcopenia muscle **(B)** Biological Process enrichment of DEGs. **(C)** Cellular Component enrichment of DEGs. **(D)** Molecular Function enrichment of DEGs. **(E)** KEGG pathway enrichment of DEGs.

Using limma and DEGseq, we identified 301 DEGs between sarcopenia and non-sarcopenia muscle samples, including 244 upregulated and 57 down-regulated genes (Figure 1A). The top five up-regulated genes were FOSB, CLLU1, FOS, EGR1, and BUB1B, whereas the top five downregulated genes were C1orf158, GOLGA6GP, MTRNR2L1, AL137246.2, and MTCO2P12. The 15 most strongly upregulated and the 15 most strongly down-regulated genes are listed in Supplementary Tables S1,S2, respectively. In total, these data identify a set of transcripts that differ between groups.

### 3.3 Functional enrichment of DEGs

GO enrichment of the 301 DEGs demonstrated significant terms across all three GO categories (Figures 1B–D). In Biological Process, overrepresented terms involved cell-cycle regulation—particularly mitotic processes—and responses to lipids (Figure 1B). In Cellular Component, enrichment localized to the nuclear lumen/nucleoplasm, cytoskeletal elements, and supramolecular complexes (Figure 1C). In Molecular Function, predominant terms included catalytic activity and ion/small-molecule/anion binding, indicating broad perturbations of enzymatic and ligand-regulated processes (Figure 1D). KEGG pathway analysis further indicated significant enrichment in canonical signaling and proteostasis pathways (Figure 1E), including the p53, MAPK, TNF, and NF-κB pathways, as well as ubiquitin-mediated proteolysis and cellular senescence.

### 3.4 Identification of inflammation-related DEGs

**FIGURE 2:**
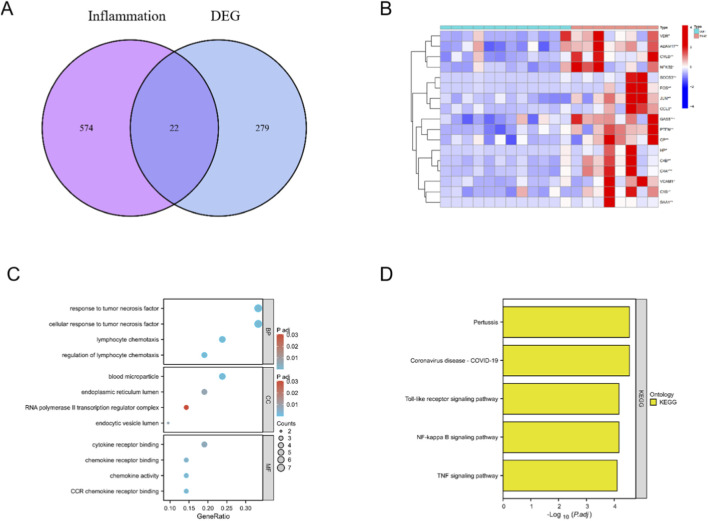
Inflammation-related gene signals distinguishing sarcopenia. **(A)** Overlap between DEGs and inflammation-associated genes identifying 22 iDEGs. **(B)** Unsupervised clustering heatmap of the 22 iDEGs separating sarcopenia from controls. **(C)** GO enrichment of iDEGs. **(D)** KEGG enrichment of iDEGs.

To probe the contribution of inflammation to sarcopenia, we queried GeneCards with the keyword “inflammation” and retained genes with a RS > 5, yielding 596 inflammation-associated genes (Supplementary Text S1). Intersecting this set with the 301 DEGs identified by DEGseq produced 22 inflammation-related DEGs (iDEGs). The overlap is depicted in a Venn diagram (Figure 2A). Unsupervised clustering of these 22 genes generated a heatmap that clearly separated sarcopenia from non-sarcopenia samples (Figure 2B).

### 3.5 Functional enrichment of iDEGs

GO enrichment analysis of the 22 iDEGs (Figure 2C) revealed significant overrepresentation of terms linked to inflammatory regulation, including response to tumor necrosis factor, lymphocyte chemotaxis, and regulation of leukocyte migration; enriched components mapped to plasma membrane–derived extracellular vesicles, the endoplasmic reticulum lumen, and RNA polymerase II regulatory complexes; and functional categories were dominated by cytokine receptor binding and chemokine activity. KEGG pathway analysis further revealed significant enrichment in canonical inflammatory cascades (Figure 2D), including TNF, Toll-like receptor, and NF-κB signaling, alongside terms linked to coronavirus disease and pertussis, which likely reflect shared innate immune and host-pathogen response modules.

### 3.6 Selection of inflammatory signature genes by random forest

**FIGURE 3:**
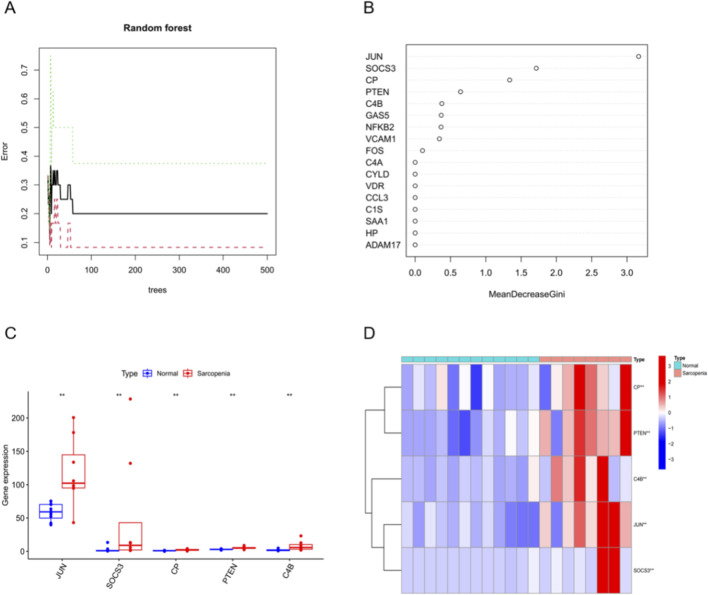
Random-forest selection and validation of a five-gene inflammatory signature. **(A)** Random-forest tuning curve showing out-of-bag error plateau at the selected hyperparameters. **(B)** Gini-based variable-importance ranking identifying JUN, SOCS3, CP, PTEN, and C4B as top contributors. **(C)** Box-and-whisker plots indicating higher expression of the five genes in sarcopenia *versus* controls. **(D)** Heatmap clustering using the five-gene panel separating sarcopenia from controls.

To derive a compact diagnostic signature, we applied a RF classifier to the 22 iDEGs. Model hyperparameters (number of trees and mtry) were tuned using OOB error, which plateaued at a stable minimum (Figure 3A). Variable importance ranked by the Gini index identified JUN, SOCS3, CP, PTEN, and C4B as the top contributors (Figure 3B). Box-and-whisker plots demonstrated consistently higher expression of these five genes in the sarcopenia group relative to controls (Figure 3C). Heatmap clustering based on the five-gene panel further separated cases from controls (Figure 3D).

### 3.7 Exploratory discrimination of the five-gene panel

**FIGURE 4:**
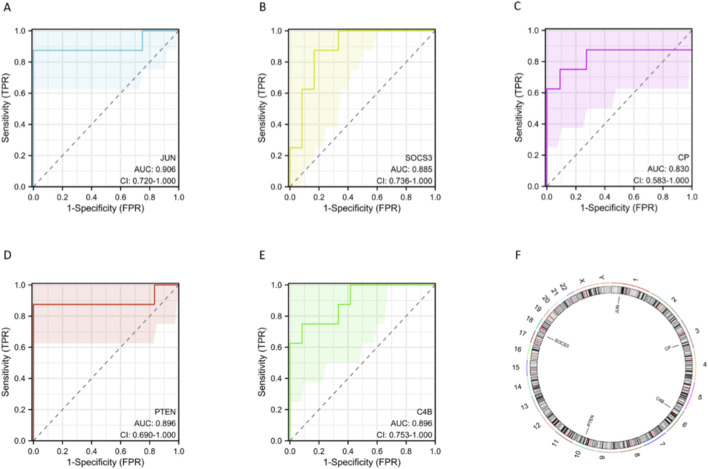
Exploratory discrimination and genomic context of the five-gene inflammatory panel. **(A)** ROC curve for JUN (AUC = 0.906). **(B)** ROC curve for SOCS3 (AUC = 0.885). **(C)** ROC curve for CP (AUC = 0.830). **(D)** ROC curve for PTEN (AUC = 0.896). **(E)** ROC curve for C4B (AUC = 0.896). **(F)** Genomic localization of the five genes shown by chord and circos plot (JUN chr1, CP chr3, PTEN chr10, C4B chr6, SOCS3 chr17).

ROC analyses indicated robust single-gene discrimination (Figures 4A–E): JUN (AUC = 0.906), PTEN (AUC = 0.896), C4B (AUC = 0.896), SOCS3 (AUC = 0.885), and CP (AUC = 0.830). These AUCs indicate apparent case–control discrimination within this dataset, with JUN, PTEN, and C4B showing particularly strong performance. Genomic localization of the five genes is illustrated in a chord and circos plot (Figure 4F): JUN (chr1), CP (chr3), PTEN (chr10), C4B (chr6), and SOCS3 (chr17), providing a genomic context for downstream interaction and pathway analyses.

### 3.8 Correlations between inflammatory signature genes and muscle phenotypes

**FIGURE 5:**
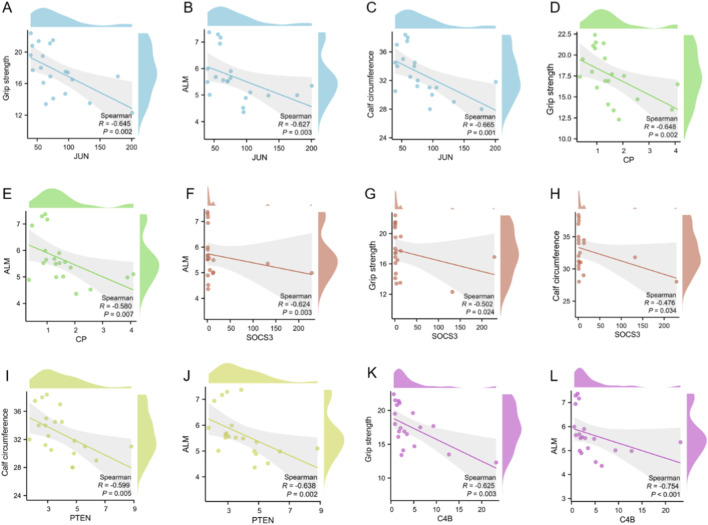
Correlations between five-gene inflammatory signature and muscle phenotypes. **(A)** JUN vs. handgrip strength (r = −0.645). **(B)** JUN vs. appendicular lean mass (r = −0.627). **(C)** JUN vs. calf circumference (r = −0.665). **(D)** CP vs. handgrip strength (r = −0.648). **(E)** CP vs. appendicular lean mass (r = −0.580). **(F)** SOCS3 vs. appendicular lean mass (r = −0.624). **(G)** SOCS3 vs. handgrip strength (r = −0.502). **(H)** SOCS3 vs. calf circumference (r = −0.476). **(I)** PTEN vs. calf circumference (r = −0.599). **(J)** PTEN vs. appendicular lean mass (r = −0.638). **(K)** C4B vs. handgrip strength (r = −0.625). **(L)** C4B vs. appendicular lean mass (r = −0.754). *P*-values are nominal (unadjusted) and should be interpreted cautiously in view of multiple comparisons.

Correlation analyses demonstrated significant negative associations between JUN expression and handgrip strength, ALM, and calf circumference (r = −0.645, −0.627, and −0.665, respectively; Figures 5A–C). CP expression was inversely correlated with handgrip strength and ALM (r = −0.648 and −0.580; Figures 5D,E), with no significant association observed for calf circumference. SOCS3 similarly showed negative correlations with ALM, handgrip, and calf circumference (r = −0.624, −0.502, and −0.476; Figures 5F–H). PTEN expression correlated inversely with calf circumference and ALM (r = −0.599 and −0.638; Figures 5I,J), whereas its association with handgrip was not significant. Finally, C4B was negatively correlated with handgrip and ALM (r = −0.625 and −0.754; Figures 5K,L), with no significant correlation with calf circumference. Together, these patterns link heightened inflammatory gene activity to poorer muscle quantity and function, reinforcing the biological plausibility of the five-gene inflammatory signature in sarcopenia. Besides, all p-values are nominal (unadjusted for multiple comparisons) and should be interpreted with caution given the number of tests.

## 4 Discussion

Age-related loss of skeletal muscle mass and function imposes a substantial burden on individuals and health systems. Early identification is essential, yet awareness and routine screening remain limited. Standard assessments of ALM are not consistently implemented, which hinders timely diagnosis and management. In this context, we aimed to identify inflammation-linked molecular markers that could support diagnosis, risk stratification, and future therapeutic development in elderly women with sarcopenia.

We acknowledge that the cohort size (n = 20) is small, which heightens the susceptibility of model-based findings to variance and potential overfitting, particularly for performance metrics such as the AUC. Consequently, the reported AUCs should be regarded as descriptive and hypothesis-generating rather than evidence of clinical performance. Confirmation in larger, independent cohorts will be required to assess robustness and generalizability.

We identified 301 DEGs in vastus lateralis biopsies, indicating broad transcriptional remodeling in sarcopenia. Among the most upregulated transcripts were FOSB, FOS, and EGR1 (see Supplementary Table S1). FOSB and FOS encode Fos family proteins within the AP-1 complex, a regulator of proliferation, differentiation, survival, and stress or inflammatory responses (Nestler, 2015; Cordier and Creytens, 2023; Shaulian and Karin, 2002). EGR1, a zinc-finger transcription factor, is rapidly induced by growth factors, injury, stress, and inflammation (Wang et al., 2021; Havis and Duprez, 2020). Although these immediate-early genes were not retained by the random-forest classifier as diagnostic features, their elevation is consistent with an activated, stress-responsive program in sarcopenic muscle that may contribute to inflammatory reprogramming.

Pathway analyses supported a multifactorial disease biology. KEGG enrichment highlighted p53, MAPK, TNF, and NF-κB signaling, together with ubiquitin-mediated proteolysis and cellular senescence, which plausibly converge on impaired myofiber integrity and regeneration. Activation of p53 in response to genotoxic or metabolic stress can promote apoptosis and senescence, limiting myogenic renewal (Engeland, 2022). Aberrant MAPK signaling may exacerbate inflammatory tone and myocyte dysfunction (Sun et al., 2015). ROS-driven TNF-α production with downstream NF-κB activation induces pro-inflammatory programs and proteolytic pathways that accelerate atrophy (Fan et al., 2016; Thoma and Lightfoot, 2018). Dysregulated ubiquitin–proteasome activity perturbs proteostasis and favors net protein loss (Gumucio and Mendias, 2013), while senescence programs impair repair capacity and niche support for muscle stem cells (He et al., 2022). Together, these results indicate that inflammatory signaling, stress responses, and proteostasis imbalance act in concert in sarcopenia.

Intersecting DEGs with inflammation-associated genes from GeneCards (RS > 5) yielded 22 iDEGs. Machine-learning feature selection prioritized a five-gene inflammatory signature (JUN, SOCS3, CP, PTEN, C4B) that separated cases from controls and showed strong single-gene diagnostic performance, with AUCs ranging from 0.83 to 0.91. Higher expression of these genes correlated with lower ALM, weaker handgrip strength, and smaller calf circumference, which supports biological plausibility.

Mechanistic interpretation of the five genes aligns with the pathway landscape above. JUN encodes c-Jun, a core AP-1 component that integrates stress and inflammatory cues to regulate survival, metabolism, and repair (Meng and Xia, 2011; Yan et al., 2018). Its negative associations with muscle quantity and strength, together with a high AUC, position JUN as a marker of inflammatory–stress activation in sarcopenic muscle. SOCS3, a negative feedback regulator of cytokine signaling, restrains JAK–STAT activation yet is induced by inflammatory cytokines; dysregulated SOCS3 may both reflect and perpetuate elevated cytokine tone implicated in sarcopenia (Gao et al., 2018; Zanders et al., 2022). CP encodes ceruloplasmin, a ferroxidase and acute-phase protein involved in iron handling and redox homeostasis (Hellman and Gitlin, 2002; Liu et al., 2022). Upregulation of CP in sarcopenic muscle may represent a compensatory response to oxidative stress and disturbed metal metabolism; prior work from our group implicating cuproptosis in sarcopenia provides a coherent link among CP expression, redox balance, and metal homeostasis (Lin et al., 2022; Tsvetkov et al., 2022). PTEN, a lipid phosphatase that antagonizes PI3K–Akt, can blunt mTOR-driven protein synthesis and favor FOXO-mediated proteolysis when upregulated, thereby promoting atrophy (Glass, 2010; Liu et al., 2021; Li et al., 2017). C4B, a component of the classical complement pathway, may indicate heightened complement activity and immune engagement within muscle tissue (Schifferli and Paccaud, 1989; Werner and Criss, 2023). Taken together, these genes capture complementary facets of inflammatory signaling, cytokine feedback, redox and metal regulation, anabolic resistance, and innate immune activation.

All participants underwent fracture surgery, and systemic CRP values were elevated in both groups without a significant between-group difference, indicating a cohort-wide, non-differential acute-phase milieu. Moreover, biopsies were obtained from the vastus lateralis, remote from the fracture site. The observed enrichment for cellular senescence, ubiquitin-mediated proteolysis, and NF-κB/TNF/MAPK signaling, together with complement activity and PI3K–Akt antagonism, aligns with established features of aging muscle and chronic low-grade inflammation. While these transcriptomic patterns are compatible with chronic, sarcopenia-related inflammation, the perioperative fracture setting with biopsies obtained approximately 2 days after injury means that residual acute-phase effects cannot be excluded; accordingly, future research could pursue external validation in ambulatory, non-trauma cohorts—and, where feasible, in non-invasive biospecimens—to evaluate generalizability.

In this pilot analysis, 15 pairwise correlations were evaluated (five genes across three muscle phenotypes), as shown in Figure 5. Because only nominal *p* values are reported without adjustment for multiplicity, the probability of false-positive findings is increased. Accordingly, the correlation results should be regarded as exploratory signals rather than definitive effects. Future studies with larger samples will prespecify primary endpoints and apply appropriate control of family-wise or false-discovery error rates to establish the robustness of these associations.

This study has limitations. Although the sample exceeded the *a priori* minimum for detecting twofold expression differences, the overall size remains modest and may limit generalizability. Given the small sample size relative to the number of predictors, there is a non-trivial risk that model performance overestimates the true discriminative ability. As such, our conclusions regarding the five-gene panel are provisional and intended to guide future validation rather than to support immediate diagnostic use. The cohort comprised elderly women, so findings may not extend to other ages or to men. Unmeasured confounders such as lifestyle and comorbidities could influence gene expression despite clinical matching. Future work should validate the five-gene signature in larger, sex-balanced, and multi-ethnic cohorts, integrate external transcriptomic datasets, and evaluate multigene models with clinically meaningful metrics such as net reclassification improvement and decision-curve analysis. Mechanistic studies that perturb JUN, SOCS3, CP, PTEN, or C4B in myotube or animal models are warranted to establish causal roles and therapeutic tractability.

## 5 Conclusion

This pilot study integrating muscle transcriptomics with machine-learning nominates an inflammation-associated five-gene panel in postmenopausal women with sarcopenia. The findings refine the pathophysiological context by linking inflammatory signaling, proteostasis, redox and metal handling, and innate immune pathways to sarcopenic muscle. Given the small cohort and surgical setting, the observed discriminative signals should be considered descriptive; confirmation in larger, independent, non-trauma cohorts is required before any appraisal of clinical utility. These results prioritize specific genes and pathways for mechanistic studies and for developing non-invasive surrogate markers in future work.

## Data Availability

The datasets presented in this study can be found in online repositories. The names of the repository/repositories and accession number(s) can be found in the article/Supplementary Material.

## Abbreviations

MuSC, muscle stem cell; DEGs, differentially expressed genes; RF, random forest; ORIF, open reduction and internal fixation; DEXA, dual-energy X-ray absorptiometry; ALM, appendicular lean mass index; RS, relevance score; OOB, out-of-bag; ROC, receiver operating characteristic; CI, confidence intervals; iDEGs, inflammation-related DEGs.
