# Supplementary material for: Inflammation centered muscle signature of sarcopenia from postmenopausal women in Shanghai China
Source: Front Cell Dev Biol. 2025 Dec 18;13:1726045. doi: 10.3389/fcell.2025.1726045 (PMC12756426; doi:10.3389/fcell.2025.1726045)
Supplement: Supplementary file 1 [file Table1.docx]

**Supplementary Table S1.** Top 15 up-regulated differentially expressed genes in elderly women with sarcopenia

| Gene id | GeneName | log2FC | pValue | qValue |
| --- | --- | --- | --- | --- |
| ENSG00000125740 | FOSB | 5.507 | 1.6E-07 | 1.4E-04 |
| ENSG00000257127 | CLLU1 | 5.504 | 8.9E-09 | 2.1E-05 |
| ENSG00000170345 | FOS | 5.247 | 8.5E-08 | 9.1E-05 |
| ENSG00000120738 | EGR1 | 5.244 | 7.5E-05 | 9.0E-03 |
| ENSG00000156970 | BUB1B | 5.217 | 7.4E-04 | 3.5E-02 |
| ENSG00000275894 | AL021578.1 | 4.877 | 3.1E-04 | 2.1E-02 |
| ENSG00000165312 | OTUD1 | 4.677 | 9.0E-04 | 3.9E-02 |
| ENSG00000184557 | SOCS3 | 4.556 | 2.5E-08 | 4.4E-05 |
| ENSG00000138180 | CEP55 | 4.549 | 5.4E-07 | 3.4E-04 |
| ENSG00000158125 | XDH | 4.476 | 1.6E-05 | 3.6E-03 |
| ENSG00000122877 | EGR2 | 4.419 | 3.4E-04 | 2.3E-02 |
| ENSG00000128016 | ZFP36 | 4.375 | 5.9E-04 | 3.1E-02 |
| ENSG00000268873 | AC022601.1 | 4.304 | 4.7E-04 | 2.8E-02 |
| ENSG00000148773 | MKI67 | 4.163 | 4.3E-08 | 5.5E-05 |
| ENSG00000257017 | HP | 4.132 | 2.5E-05 | 4.6E-03 |

**Supplementary Table S2.** Top 15 down-regulated differentially expressed genes in elderly women with sarcopenia

| Gene id | GeneName | log2FC | pValue | qValue |
| --- | --- | --- | --- | --- |
| ENSG00000157330 | C1orf158 | -5.211 | 1.0E-07 | 9.9E-05 |
| ENSG00000261303 | GOLGA6GP | -5.156 | 4.1E-04 | 2.6E-02 |
| ENSG00000256618 | MTRNR2L1 | -4.231 | 6.8E-07 | 4.0E-04 |
| ENSG00000278445 | AL137246.2 | -2.395 | 1.1E-03 | 4.4E-02 |
| ENSG00000229344 | MTCO2P12 | -2.329 | 2.0E-04 | 1.7E-02 |
| ENSG00000235070 | AC062015.1 | -2.271 | 4.2E-05 | 6.1E-03 |
| ENSG00000232177 | MTND4P24 | -2.261 | 6.6E-05 | 8.4E-03 |
| ENSG00000225972 | MTND1P23 | -2.258 | 1.1E-03 | 4.2E-02 |
| ENSG00000186326 | RGS9BP | -2.173 | 2.8E-08 | 4.6E-05 |
| ENSG00000164161 | HHIP | -2.135 | 8.3E-05 | 9.6E-03 |
| ENSG00000137675 | MMP27 | -2.096 | 9.0E-04 | 3.9E-02 |
| ENSG00000214097 | SMCO1 | -2.078 | 1.4E-03 | 4.9E-02 |
| ENSG00000185669 | SNAI3 | -2.048 | 8.0E-09 | 2.1E-05 |
| ENSG00000075702 | WDR62 | -1.949 | 2.8E-04 | 2.0E-02 |
| ENSG00000187942 | LDLRAD2 | -1.904 | 3.2E-06 | 1.1E-03 |
